# An Update on Human Papilloma Virus Vaccines: History, Types, Protection, and Efficacy

**DOI:** 10.3389/fimmu.2021.805695

**Published:** 2022-01-27

**Authors:** Zahra Yousefi, Hamid Aria, Farhoodeh Ghaedrahmati, Tahereh Bakhtiari, Mahdieh Azizi, Reza Bastan, Reza Hosseini, Nahid Eskandari

**Affiliations:** ^1^ School of Allied Medical Sciences, Shahroud University of Medical Sciences, Shahroud, Iran; ^2^ Department of Immunology, Faculty of Medicine, Isfahan University of Medical Sciences, Isfahan, Iran; ^3^ Department of Immunopharmacology, Faculty of Medicine, Karaj University of Medical Sciences, Alborz, Iran

**Keywords:** human papillomavirus (HPV), vaccines, HPV vaccination, cervical cancer, prevalence

## Abstract

Human papillomavirus (HPV) is the most common sexually transmitted agent worldwide. Early prevention with HPV vaccination is a safe and effective method against this disease. HPV vaccines provided more protection against several oncogenic HPV strains. Three prophylactic HPV vaccines have been approved to target high-risk HPV types and protect against HPV-related disorders. These existing vaccines are based on the recombinant DNA technology and purified L1 protein that is assembled to form HPV empty shells. The prophylactic vaccines are highly immunogenic and can induce production of specific neutralizing antibodies. However, therapeutic vaccines are different from these prophylactic vaccines. They induced cell-mediated immunity against transformed cells, instead of neutralizing antibodies. The second generation of prophylactic HPV vaccines, made from alternative viral components using cost-effective production strategies, is undergoing clinical evaluation. The purpose of this review is to provide a complete and up-to-date review of the types of HPV vaccines and the efficiency of each of them for readers.

## Introduction

Vaccination is a low-cost and effective method to reduce the risk of infectious diseases. Implementing a universal vaccination program significantly controlled and eradicated many infectious diseases and profoundly affected human health. Currently, there are effective human vaccines against different pathogens such as viruses, which induce various disorders and kill tens of millions of people each year. The human papillomavirus (HPV) is a virus that affects the different parts of the body. Genital HPV infection is the most common sexually transmitted infection worldwide, involving 75% to 80% of men and women of all ages (1). The HPV virus can stay in the skin and develop a genital wart. Genital warts are small bumps or growths that appear on the genitals and increase the risk of developing cervical cancer. There are many types of HPV vaccines. All HPV vaccines can protect against high-risk HPV types, including HPV16 and HPV18 that cause most HPV cancers (2–4). This review is a comprehensive study on the available HPV vaccines and their efficiency.

HPV has been recognized as the cause of around 5% of all cancers worldwide (5). For the first time, Dr. H. zur Hausen of the University of Heidelberg in Germany, on Feb. 12, 1985, introduced HPV. The report said that “finding strong evidence linking viruses in the family called papilloma with genital cancers, notable cancers of the cervix and vulva (6).” Papillomaviruses are a large group of nonenveloped double-stranded DNA viruses that constitute the papillomavirus genus of the Papillomaviridae family and infected humans. There are more than 100 types of HPV, which have been subdivided into cutaneous or mucosal categories based on their tissue tropism (7). In addition, HPV types have been grouped into high risk (HR) and low risk (LR) according to their oncogenic capacity (8).

## Epidemiology and Prevalence of HPV

HPV16 and HPV18 are among the most common and high-risk types of HPV that can be controlled using vaccination (9). A meta-analysis in of 157,879 women, with normal cervical cytology, demonstrated that point prevalence of HPV is approximately 10% worldwide (10). A higher HPV infection prevalence was found in Africa with 22% of women being found positive for HPV infection. The prevalence of cervical HPV infection decreases more rapidly in women after the age of 30 years (11). Women with persistent infection have the highest risk of developing high-grade squamous intraepithelial lesions or invasive cervical cancer. In addition, longitudinal studies have demonstrated that cervical HPV infection increased the prevalence of anal HPV infection diseases (11).

However, the epidemiology of HPV among men is different when compared with the female populations. The most prevalent reported factors that are associated with HPV infection in men include human immunodeficiency virus (HIV) infection, sexual behavior in the current and past years, number of sex partners, not using a condom, race, ethnicity, and circumcision status (12–16). The natural history of HPV infection has indicated lower rates of HPV clearance in uncircumcised men compared with circumcised men (16). A systematic analysis of articles published from 1990 to 2006 reported that the prevalence of HPV in men ranged from 1% to 73% (17). This wide range of prevalence rates is attributed to various factors including the number of processed specimens, anatomic sampling sites, and of detection methods. These results are consistent with the findings of demographic analysis that demonstrate an association between increased sexual activity and genotypes of high-risk HPVs (7). Epidemiological studies have revealed that HR types are associated with cervical intraepithelial neoplasia (CIN), invasive cervical cancer, and its precursor lesion in women, whereas HR subtypes are causes of head and neck squamous cell carcinoma and penile cancer in men (17, 18).

In addition, studies revealed a bidirectional relation between epidemiology and prevalence of two viruses, namely HPV and HIV; HPV infection is more common among HIV-positive patients than uninfected persons (7).

## Pathogenesis of HPV Infection

HPV are small, nonenveloped and double-stranded DNA viruses that infect both mucosal and cutaneous epithelial cells. The DNA genome of HPV encodes about eight open-reading frames (ORFs). The ORFs are divided into three functional regions, including the early (E) region, late (L) region, and noncoding part or long control region (LCR). The genes of the E region encode proteins E1–E7 that are necessary for viral replication and involved in the pathogenicity of the virus. The genes of the L region encode capsid proteins L1 and L2 required for virions assembly. In addition, LCR genes are important for the replication and transcription of viral DNA and have a tropism for epithelial cells. HPV can infect epithelial cells *via* interaction with cell surface receptors such as integrin α6, which are abundantly expressed in the basal cells and epithelial stem cells (19). Consequently, a virus with a low copy number may infect primitive basal cells. Shortly after localized infection and viral DNA replication, the number of viruses increases to approximately 50–100 copies per cell (20). E1 and E2 proteins are both required to initiate papillomavirus DNA replication. First, E1 as a dimer along with dimer of E2 binds to the viral origin, leading to the assembly of E1–E2 ternary complex that blocks nonspecific interaction of E1-DNA. This complex acts as a template for the recruitment of additional molecular binding of E1 and E2 and assembly of the E1 double-trimer intermediate. Ultimately, double-hexameric E1 helicases with ATPase activity assemble, which are capable of DNA unwinding and engaging with the cellular DNA replication factors (21). The expression of viral genes is minimal in the phases of plasmid or episomal maintenance. The expression of several viral oncogenes such as E6 and E7 proteins is highly controlled during the normal life cycle of HPV.

**Figure 1:**
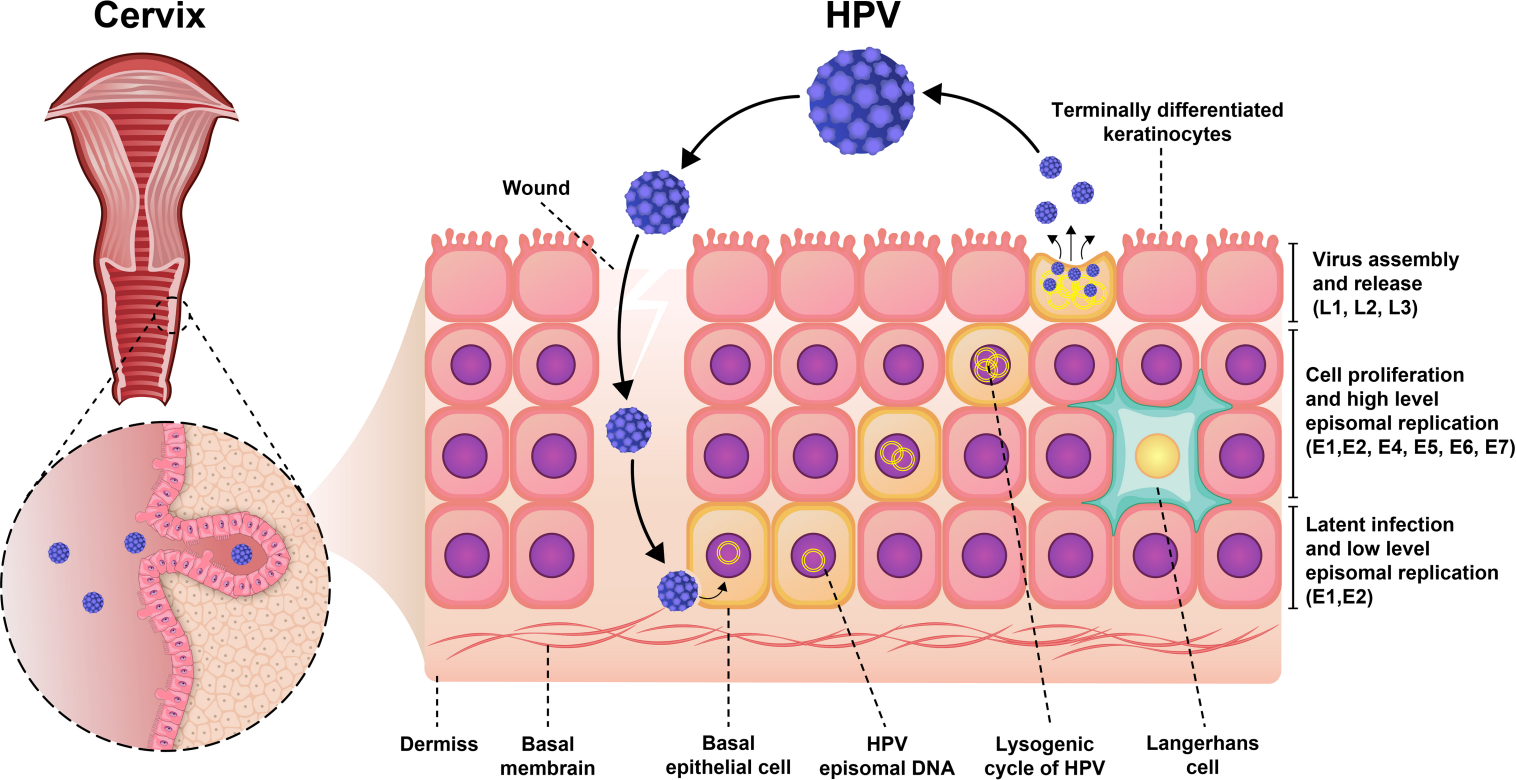
Pathogenesis of HPV infection. Initially, the virus was latent inside the epithelial cell and had a low proliferation rate. As the virus enters the lysogenic cycle, the rate of proliferation increases. Finally, the viruses are assembled and secreted from keratinocytes to repeat the infection cycle.

Moreover, the expression of viral genes is highly upregulated when the infected cells enter the different—and cell proliferation—compartments of the cells (**Figure 1**). During viral DNA replication, there are at least 1,000 copies of the virus per cell, and these virions increase the expression of L1 and L2 capsid proteins along with the assembly of infectious viruses (20, 22). The life cycle features of HPV play an important role in the evading recognition by the immune system and viral pathogenesis. The life cycle of HPV is characterized by nonlytic immunity of infected cells and the lack of viremia and inflammatory signals (23).

## Immune Response to HPV

The low-risk oncogenic types of HPV could cause cervical, vaginal, vulvar, and anal cancers in women and penile, anal, and oropharyngeal cancers in men. The low-risk nononcogenic types of HPV are linked to warts and other benign pathologies in both sexes. Fifteen HPV including HPV16, HPV18, HPV31, HPV33, HPV35, HPV39, HPV45, HPV51, HPV52, HPV56, HPV58, HPV59, HPV68, HPV73, and HPV82 are thought to be high risk (23). In most patients, HPV infection is asymptomatic and can be controlled by the immune system. However, in cases with specific conditions such as people with immune system disorder, older age, and multiple partners, latent viral reactivation occurs following primary HPV infection. In a small number of cases, the viral lesions progress to invasive cancer, especially when HPV types 16 and 18 are involved (21). Most sexually active men and women will be infected with one or more types of HPV at some point in their lives, but the majority of them will improve from infection without any symptoms (23). The immune responses play a crucial role in clearing most HPV infections. Two major parts of the immune system, the innate immune system and the adaptive immune system, developed against HPV infections. During any microinjury from a bacterial infection or sexual contact, antigen-presenting cells (APCs) are exposed to HPV proteins. The Langerhans cells (LCs), which are highly specialized APCs, presented the HPV proteins in the epidermis. However, LCs fail to induce a sufficient immune response against HPV16 L1, leading to immune tolerance (24). Monocyte-macrophage and dendritic cells (DCs) in the skin are key players in recognizing HPV antigens when HPV infects the epithelium. These cells induce the effector immune responses by releasing important proinflammatory cytokines, including interleukin (IL)-1, IL-6, tumor necrosis factor alpha (TNF-α), and IL-12, which stimulate local inflammation and serve as danger signals. This process is essential for inducing adaptive immune responses (25). The innate immune responses are only useful in the early clearance of viral infection, and adaptive immune responses are necessary to regression-established lesions. Several studies revealed that CD8+ cytotoxic T cells and CD4+ T helper 1 (TH1) cells (which produce IL-2 and IFN-γ) with recognition of E2 and E6 HPV proteins play an important role in the clearance of low-grade HPV infection. However, CD4+ T cells with recognition of E7 proteins control the high-grade neoplasia (24, 26, 27). Because of the low level of virus titers in the basal lamina and the production of newly assembled HPV virions in the upper epithelial layers, an inadequate humoral immune response has been reported during natural HPV infection (28). IgG and IgA are the most abundant antibody isotypes detected in sera of patients with natural infections.

**Figure 2:**
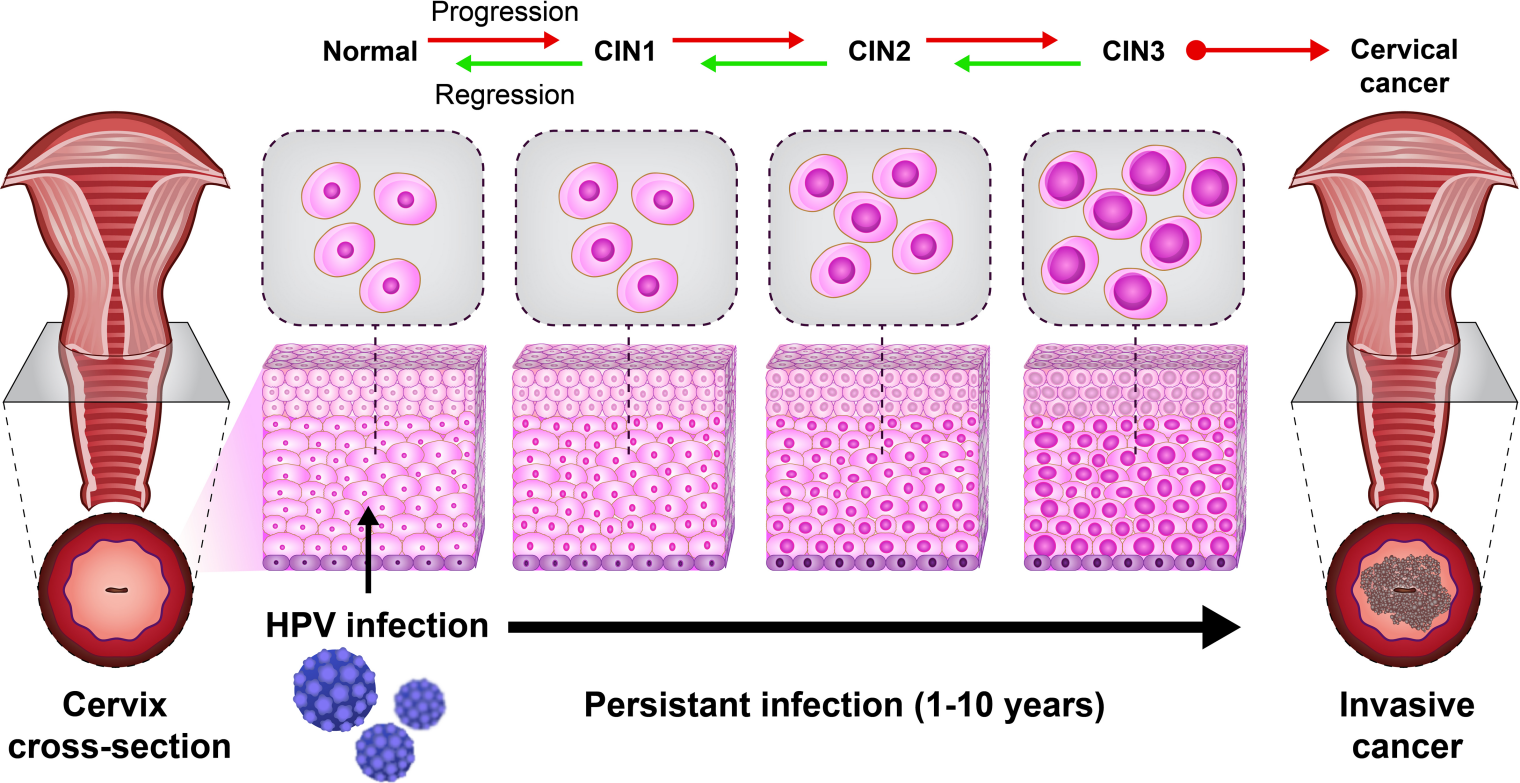
The natural HPV infection can lead to cervical cancer. The cervical intraepithelial neoplasia (CIN) scale is classified into CIN1 (mild dysplasia), CIN2 (moderate dysplasia), and CIN3 (severe dysplasia and carcinoma *in situ*).

A previous study revealed the role of immune cells in the induction of CIN. CIN is a potential premalignant transformation and dysplasia of cervical squamous cells, mainly caused by high-risk HPV types 16 and 18 (29). The CIN is classified into CIN1 (mild dysplasia), CIN2 (moderate dysplasia), and CIN3 (severe dysplasia and carcinoma *in situ*). A lesion may be asymptomatic and regress spontaneously or progress to invasive cancer (21) (**Figure 2**). Histological regression and immune response represent an association between CIN1 with the granzyme B expression of CD8+ T cells and CD56+ NK cell intra lesions. Also, immunohistochemically, studies report that CD8+ T cells in CIN1 and koilocytic cells of cervical lesions expressed α4/β7 integrin (25).

## Immune Evasion Strategies of HPV

HPV uses a variety of strategies to the downregulation of immune responses. These evasion mechanisms facilitate disease progression from infection to cancer (30). HPV can evade recognition by immune cells using low-level expression of viral antigens (30). Also, the production of virions only in the outermost layers of epithelial and not lyse the infected cells limited the access of immune cells to HPV (31). HPV altered gene expression of the host cells using perturbation of DNA methylation. Ultimately, DNA methylation leads to the downregulation of important mediators of immune responses such as chemokines, adhesion molecules, and Toll-like receptors (TLRs) (32). HPV disrupts the function of host proteins through protein-protein interaction (33). The interferon pathway disrupted in HPV-infected keratinocytes by the E5, E6, and E7 binding to interferon response factors (IRFs), which are transcription factors for interferon-induced genes (34). It has been reported that the expression of cyclic guanosine monophosphate-adenosine monophosphate synthase-stimulator of interferon genes (cGAS-STING) as an important defense mechanism against DNA viruses is dysregulated by HPV18 (35). Infected HPV16 cells decrease the expression of immunoproteasome subunits PSMB8 and PSMB9 involve in antigen processing (36). HPV downregulates the cell-surface expression of major histocompatibility complex class I (MHC-I) by E5 oncoprotein through retention in the Golgi complex (37–39). Taken together, the downregulation of immune response by the oncoproteins of HPV allows the infected cells to evade the immune system cells and facilitate the persistence of virus infection (30).

## HPV and Etiology of Human Cancers

HPV as the most common sexually transmitted infection can progress to invasive cervical cancer. Approximately 10%–20% of individuals with a persistent cervical HPV infection showed a higher risk of CIN2/3 lesion (40). The evasion mechanisms of HPV from the immune cell recognition and downregulation of innate and adaptive immune responses lead to the establishment and persistence of HPV infection (25, 41). The oncoproteins E5, E6, and E7 play a crucial role in the malignant transformation of HPV-related lesions. The formation of tetraploid cells using induction of cell fusion and failure of cytokinesis are key functions of E5 protein in HPV infection and tumor progression. The role of E6 to induce HPV-related malignancies is associated with the formation of a complex ubiquitin ligase UBE3A and induces p53 ubiquitination. The tumor suppressor p53 is controlled by ubiquitination and degradation of 26S proteasome (21). In addition, E7 protein regulates the cell cycle progression *via* different mechanisms, including interaction with p21 and p27, driving the entry of cells to S-phase, and inactivating tumor suppressor protein retinoblastoma. The limited replication of viral episome in CIN1 and some CIN2 lesions decreases the expression levels of E6 and E7 proteins in persistent HPV infection. However, high E6 and E7 expression levels have been detected in some CIN2 lesions, CIN3, and invasive cancers, whose viral DNA is integrated into the host cell genome (23). These results strongly indicate that oncoproteins E5, E6, and E7 have major roles in the negative regulation of immune response and development of HPV-associated cancers.

## HPV and Vaccination

The use of vaccines that activate cytotoxic cells and stimulate cell immunity responses effectively prevents viral infection. There are different vaccines against viral infections, such as prophylactic and therapeutic vaccines (28, 42). Studies have shown that most viral vaccines have been used successfully for vaccination-induced humoral immune responses (43). Similar results were obtained from prophylactic HPV vaccines, showing protection against persistent infections and premalignant neoplasia by inducing neutralizing antibodies (mostly IgG). Therapeutic vaccines are different from prophylactic vaccines. They stimulate cell-mediated immunity (especially CD8+ T cells) rather than neutralize antibodies. To date, no therapeutic vaccines are approved for use in viral infection. Although, various researcher teams are trying to develop a safe and effective therapeutic vaccine (44). E6 and E7 genes are the optimal targets for therapeutic vaccination because they are expressed continuously in malignant tissues and are involved in cell cycle arrest. Different methods have been investigated for the synthesis and development of therapeutic vaccinations, including nucleic acid-based, peptide-based, protein-based, cell-based, and live-vector vaccines, all of which are currently in clinical trials. Clinical trials of HPV therapeutic vaccines show that they are safe and efficient in treating cervical cancer while also having limitations. A bacterial vector vaccine, ADXS11-001, and a DNA vaccine, VGX3100, are both in phase III clinical trials, indicating that they have promising potential (45). pNGVL4aCRT/E7 (NCT01493154) (46), VGX-3100 (NCT01304524) (47), and GX-188E (NCT02139267) (48) are based on E6/E7 gene vaccination against HPV and used electroporation as a delivery technique. Many clinical trials are now looking into the role of checkpoint inhibitor therapy. The combination of a therapeutic HPV vaccine and anti-PD1 therapy has been proven to be effective so far (49). It is critical to design various platforms for simultaneous use in order to prevent the restrictions of each platform. By reducing T-cell inhibition and enhancing proinflammatory cytokines, combination therapy may solve some of the potential drawbacks of therapeutic vaccinations. Peptide-based vaccines, for example, can be used as a booster for viral-based vaccinations to prevent antivector immunity (NCT03911076) (50).

Reductions in the prevalence and incidence of HPV, genital warts, and cervical lesions were seen in regions with HPV vaccination coverage. These decreases were seen also in unvaccinated females and males, indicating that herd immunity was in effect (51). When used in HPV-negative young women lower than 25 years old in a three-dose regimen, the efficacy of HPV vaccines is approximately 100%. There was no link between HPV vaccination and major adverse effects, according to data in immunization programs that have already administered over 270 million doses (52).

## Prophylactic Vaccines

The prophylactic vaccines activate the humoral immunity and production of virus-neutralizing antibodies, inhibit viruses from entering into host cells, and induce effective protection against HPV infection. To date (2021), three prophylactic licensed vaccines for the prevention of high-risk HPV infection are available in most countries: the vaccinations are Gardasil, Cervarix, and Gardasil-9. These vaccines were produced by recombinant DNA technology using the HPV L1 capsid proteins, which self-assembled into the noninfectious form of virus-like particles (VLPs). The VLPs contain no viral DNA genome and no live HPV, which is noninfectious and nononcogenic. The first generation of prophylactic vaccines was approved in 2006 and named Gardasil™ (Merck, West Point, PA, USA) or quadrivalent human papillomavirus recombinant vaccine (53). It has VLPs containing low-risk HPV6 (20 μg) and HPV11 (40 μg) and high-risk HPV16 (40 μg) and HPV18 (20 μg), which are responsible for 90% of genital warts. Cervarix™ (GlaxoSmithKline, Rixensart, Belgium) or human papillomavirus bivalent vaccine recombinant contains VLPs of high-risk HPV16 (20 μg) and HPV18 (20 μg), which cause approximately 70% of invasive cervical cancers worldwide (54). This vaccine was approved in 2009. The bivalent vaccines were produced using baculovirus-infected insect cell Trichoplusia, and the quadrivalent vaccines were made by Saccharomyces cerevisiae that expressed the L1 gene. To improve the efficacy of immune responses for a longer time, the bivalent vaccines contain the proprietary adjuvant ASO4, which is constructed from 500 mg aluminum hydroxide and 50 mg toll-like receptor 4 agonists, 3-*O*-desacyl-4′ monophosphoryl lipid A as an additional immunostimulator. In addition, quadrivalent vaccines used 225 mg amorphous aluminum hydroxyphosphate sulfate (AAHS) as an adjuvant. It has been reported that both vaccines revealed effective safety and immunogenicity profiles. However, bivalent vaccines produced higher immunogenicity against HPV infection than quadrivalent vaccines (55). The second generation of prophylactic anti-HPV vaccine is Gardasil-9™ (Merck, West Point, PA, USA) or human papillomavirus 9-valent recombinant vaccine. It has VLPs for two low-risk HPV6 (30μg) and seven high-risk HPV11 (40 μg), HPV16 (60 μg), HPV18 (40 μg), HPV31 (20 μg), HPV33 (20 μg), HPV45 (20 μg), HPV52 (20 μg), and HPV58 (20 μg). This vaccine is a nonavalent anti-HPV vaccine and was approved in 2014. The oncogenic HPV subtypes 31, 33, 45, 52, and 58 cause more than 15% cervical cancer. In addition, the nonavalent vaccine developed using Saccharomyces *cerevisiae* express L1 gene and 500 mg AAHS as an adjuvant (56, 57). The nonavalent vaccine is available for use in the USA and taken before sexual activity (58). All three anti-HPV vaccines are administered in the intramuscular route. HPV vaccines are recommended for use in girls 11–12 years old and women with immunosuppressed or immune systems deficiency. Although the efficiency of anti-HPV vaccines was initiated and confirmed after a three-dose administration in women aged 16–25 years, the Advisory Committee on Immunization Practice (ACIP) in 2016 endorsed that only two doses (ranged by 6–12 months) of vaccination are needed for persons less than 15 years of age (59). However, for immunocompromised women or started vaccination between 15 and 45 years, a three-dose program (at 0, 1–2 months, 6 months) is recommended (59–61). In addition, the third dose of vaccine should be undertaken for individuals who do not receive the vaccine before the aged of 15 years. Also, vaccination of 15- to 26-year-old women, 15- to 21-year-old men, and high-risk men up to 26 years of age is recommended in a three-dose series (61, 62). The HPV vaccines are safe, and their local adverse reactions such as pain, swelling, and redness are usually short and reversible. The systemic reactions of the available HPV vaccines, including fever, nausea, dizziness, fatigue, headache, and myalgia are rarely observed after vaccination (63). Several studies revealed that HPV VLPs induced an effective humoral immune response, and HPV vaccination generates 10- to 100-fold higher titers of specific neutralizing antibodies against HPV antigens than natural infection (64–66). However, the levels of produced antibodies are dependent on the sex and age of vaccinated individuals and the type of administrated vaccine. The comparison of seroconversion in males and females aged under 30 years demonstrated higher titers of specific antibodies among females ages 9 and 15 years vs. females who had the vaccine at 16 and 26 years old (67–71). Long-time evaluation of the immunogenicity of HPV16/HPV18 vaccine in the serum of females aged between 15 and 55 years showed high seropositive antibody levels for anti-HPV16 vaccine in all age groups, 10 years after the first dose of vaccination. In contrast, seropositivity rates for anti-HPV18 decreased in the age group of 15–55 years with aging (67). However, anti-HPV16 and anti-HPV18 antibodies were higher than natural infection with HPV in all studied groups and could be detected more than 30 years after vaccination (72). In addition, the use of adjuvant and the total number of administrated doses could affect the immunogenicity of HPV vaccines. In some of the vaccines, such as bivalent vaccines, addition of adjuvants improved the magnitude and durability of immune responses (65, 73). Moreover, the active component of HPV vaccines can affect the efficiency of immune response and the levels of neutralizing antibodies. The concentration of each L1 VLPs and the ratio of antigen to adjuvant are important differences between different prophylactic HPV vaccines, including Gardasil and Ceravix. Gardasil has twice the concentrations of HPV16 L1 VLP and an equivalent concentration of HPV18 L1 VLP compared with Cervarix. Gardasil-9 contains twice the amount of HPV18 L1 VLP, 50% more HPV16 antigen, and twice the amount of adjuvant in Gardasil. The studies revealed a relationship between the immunogenicity of HPV vaccines and the number of vaccination doses. Two doses of the HPV vaccine are more protective than one, but different studies have not detected statistically significant differences between two and three doses (59, 65, 73). After three administration doses, these findings demonstrated that HPV vaccines could induce a higher antibody response in younger girls than older girls.

The types of prophylactic HPV vaccines elicited specific immune responses, but the structural similarity between L1 genes of vaccine and nonvaccine HPV types led to long-term cross-reactive immunogenicity against HPV types not included in the vaccine. Previous studies have been reporting a cross-protection against HPV31 and HPV45 types after administration of bivalent (HPV16/HPV18) vaccines. In addition, cross-reactive immunogenicity has been detected against HPV45 for the quadrivalent HPV vaccine (74). These findings suggest that HPV vaccination could induce an effective immune response against nonvaccine HPV types.

**Figure 3:**
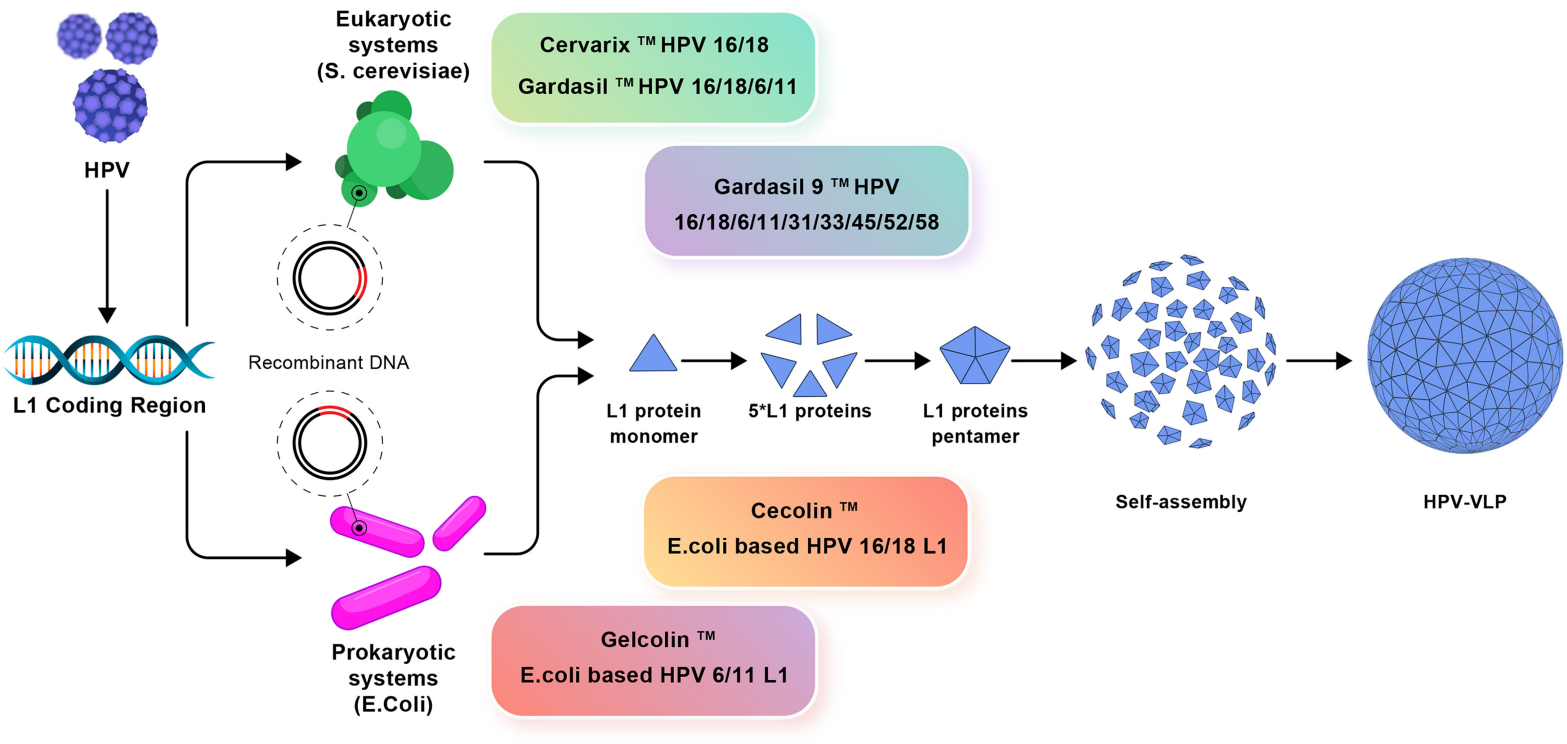
The production mechanisms of prophylactic vaccines. Eukaryotic systems including Cervarix, Gardasil, and Gardasil-9 vaccines and prokaryotic systems including Cecolin and Gelcolin vaccines used against HPV and how to produce VLP are shown.

In recent years, various bacteria such as *Escherichia coli* (*E. coli*) have been used to produce HPV L1 VLP vaccine instead of expensive eukaryotic systems (75). Two *E. coli*-derived VLP-based vaccines against HPV16/HPV18 [*E. coli*-based HPV16 and HPV18 L1 VLPs (Cecolin™)] and HPV6/HPV11 [*E. coli*-based HPV6, HPV11 L1 VLPs (Gelcolin™)] are currently in phase III and phase I clinical trials, respectively (**Figure 3**) (76). In addition, to reduce the production costs of HPV vaccines, the methylotrophic yeasts *Pichia pastoris* and *Hansenula polymorpha* are used, as suitable expression systems, for the production of HPV6, HPV11, HPV16, and HPV18 VLPs. The *Pichia pastoris*-based HPV16 and HPV18 VLPs as well as *Hansenula polymorpha*-based HPV6, HPV11, HPV16, and HPV18 VLPs, are in phase I clinical trials (76). In parallel, advanced systems consist of transgenic plants and attenuated bacteria (e.g., mutant *Salmonella enterica* or *Shigella*) capable of producing L1 VLPs (77).

Although capsid protein L1 is highly immunogenic and used to produce current HPV vaccines, the minor capsid protein L2 that is highly conserved among the different HPV types could be used as an appropriate candidate for the production of pan-HPV vaccine (75). In addition, this protein can be obtained by bacteria (78). However, L2-based vaccines produced lower neutralizing antibody levels than L1-protein-based VLPs. Strategies to improve the immunogenicity of L2 protein have been evaluated and revealed hopeful and promising results. Chimeric L1–L2 virus-like particles produced based on the high immunogenicity of L1-based vaccines and the wide cross-protection of L2 could increase the therapeutic potential of current HPV vaccines (79). To date, explanatory studies have been performed to produce effective HPV vaccines using various components of prophylactic HPV vaccines, such as a combination of L2 protein and early HPV proteins E6 or E7. L2 VLP is less immunogenic than L1 VLP and can elicit long-lasting neutralizing antibody responses as well as protection against a variety of HPV strains. A display of L2 peptides on bacteria, viral capsids, or VLPs and other platforms are examples of techniques to improve the strength of the humoral response. L2-based vaccines have no therapeutic potential on their own, but when combined with viral oncoproteins, E6 and E7, they may generate both preventive immunity and therapeutic immunity (80). Even without adjuvant, mice inoculated with a single dose of MS2–16L2 VLPs were partially protected against challenge with a high dose of HPV16 1 year later (81). A vaccination based on L2 also has the potential to lower the incidence of cutaneous squamous cell malignancies (82). In addition, there is a great interest in developing combined prophylactic and therapeutic vaccines. The components of various prophylactic HPV vaccines are listed in **Table 1**.

**Table 1:** Various types of prophylactic HPV vaccines.

	Cervarix	Gardasil, Silgard	Gardasil-9
**Valency**	2-Valent	4-Valent	9-Valent
**Types**	HPV16/18	HPV6/11/16/18	HPV6/11/16/18/31/33/45/52/58
**Adjuvant**	ASO4 (0.5 mg aluminum hydroxide and 50 µg 3-*O*-desacyl-4″-monophosphoryl lipid A (MPL))	0.225 mg aluminum hydroxyphosphate sulfate	0.5 mg aluminum hydroxyphosphate sulfate
**Expression system**	Baculovirus-insect cell	Yeast	Yeast

The TA-GW vaccine is on alum made up of a fusion protein containing the L2 and E7 proteins of HPV6 produced in *E. coli*. Immunogenicity was demonstrated in T-cell and antibody responses in phase I investigations in healthy volunteers and a phase IIa clinical study in 25 genital wart patients (83, 84). Five individuals in the phase IIa study were completely free of warts after 8 weeks. There were no recurrences of warts in any of the 13 people whose warts were eradicated by the vaccine. 2A Pharma conducted a phase I clinical trial (NCT03929172) in healthy adult to examine the safety of AAVLP vaccine and its immunogenicity against HPV16/31. The outcomes of this experiment, as the most advanced attempt to develop an L2-based HPV vaccination, are enthusiastically expected.

### Efficacy and Immunogenicity of Prophylactic Vaccines

The bivalent and quadrivalent HPV vaccines provide high levels of protection against persistent HPV16 and HPV18 infection. Trials evaluating the mono-, bi-, and quadrivalent prophylactic HPV vaccines revealed that vaccines produce lower levels of neutralizing antibodies in women vaccinated at age 24 to 45. Accordingly, young women aged 15 to 26 years are the primary target population to receive HPV vaccines (85).

In addition, previous research showed that prophylactic vaccine Cervarix had more protection than Gardasil against HPV infection (86). The prophylactic HPV vaccines play a crucial role in preventing various disorders caused by HPV through the production of specific antibodies. Therefore, the levels of neutralizing antibodies are used to evaluate the immunogenicity of these vaccines.

The clinical research showed similar immunogenicity against HPV infection and cervical cancer after administering three doses of different prophylactic vaccines. However, the levels of anti-HPV16 and anti-HPV18 antibodies were significantly lower after Gardasil administration compared with Cervarix (87). The findings of three serological assays including pseudovirus neutralization assay (WHO suggests), competitive immunoassay (epitope-specific), and 3-VLP-IgG binding assays, revealed that a single-dose administration of Gardasil and Gardasil-9 induced low levels of antibodies, especially against HPV18 and HPV45 (88, 89).

The evaluation of immune response to HPV, 1 year after vaccination with the AS04-adjuvanted HPV-16/HPV18 vaccine, revealed the specific IgG and IgA antibodies in serum of vaccinated individuals. One month after vaccination, the levels of serum IgA were 95% and decreased with time. The levels of IgA were reported at 79% a year after vaccination. Similar results were observed for levels of serum IgG antibody. These results suggest that a booster vaccine dose and the use of suitable adjuvants can improve the immune responses and increase the levels of neutralizing antibodies (90).

## Challenge and Barriers of HPV Vaccines

From June 2006, when the HPV vaccines were first approved in the USA, numerous real-world data have demonstrated the safety and effectiveness of the HPV vaccination program to prevent and treat HPV infection and related diseases. However, there are multiple barriers, including high vaccine costs, inaccessibility, and lack of suitable storage or transportation conditions (91). In addition, there is no public knowledge about HPV-related disorders and national vaccination programs in most low- and middle-income countries (92). Vaccination campaigns must provide accurate information about safety, efficacy, and healthcare professionals of HPV vaccines differently (91). The main challenges of implementing HPV vaccines are that the vaccines do not protect against all types of HPV (93).

## Conclusions

HPV is a common virus that can be easily transferred from person to person. Some types of HPV cause different cancers. It is essential to know how to prevent HPV infection or HPV-related cancers. Vaccination is an effective method for the early prevention of HPV infections. There are safe and highly effective prophylactic vaccines to prevent many HPV-related disorders. The bivalent and quadrivalent HPV vaccines appear to be significantly effective in preventing HPV infection after introducing vaccines into vaccination schedules. Bivalent vaccines produced higher immunogenicity against HPV infection than quadrivalent vaccines. A booster vaccine dose and adjuvants can improve the immune responses through neutralizing antibodies. Young women aged 15 to 26 years showed higher levels of neutralizing antibodies so, they are the primary target for receiving HPV vaccines. Recent studies confirmed that administration of nonavalent HPV vaccine before starting sexual activity provides effective protection against multiple HPV subtypes. Recent studies of HPV vaccines tend to focus on the production of vaccines based on L1 and L2 capsid proteins in live viral or bacterial vectors, with cost-effective production systems. In addition, combined prophylactic and therapeutic vaccines could be prevented and treat HPV-related diseases. The next generation of HPV vaccines will reduce many limitations related to available vaccines and is a major step toward the fight against cervical cancer. Soon, developed vaccines will likely generate good protection against various types of HVP, being based on recombinant vectors, and maybe administrated by inhalation or through oral route. However, further clinical investigation is required to develop and validate cost-effective new vaccines with proper immunogenicity against various types of HPV. Understanding the specific pathological mechanisms of HPV is helpful in the development of more effective vaccines that are frequently used in the clinic.

## Author Contributions

ZY, HA, FG, TB, MA, RH, and NE conceptualized the study and wrote the manuscript. RB contributed to drafting of the manuscript. All authors listed have made a substantial, direct, and intellectual contribution to the work and approved it for publication.

## Conflict of Interest

The authors declare that the research was conducted in the absence of any commercial or financial relationships that could be construed as a potential conflict of interest.

## Publisher’s Note

All claims expressed in this article are solely those of the authors and do not necessarily represent those of their affiliated organizations, or those of the publisher, the editors and the reviewers. Any product that may be evaluated in this article, or claim that may be made by its manufacturer, is not guaranteed or endorsed by the publisher.
